# LMFD: lightweight multi-feature descriptors for image stitching

**DOI:** 10.1038/s41598-023-48432-7

**Published:** 2023-11-30

**Authors:** Yingbo Fan, Shanjun Mao, Mei Li, Jitong Kang, Ben Li

**Affiliations:** Institute of Remote Sensing and Geographic Information Systems, Peking University, No.5 Summer Palace Road, Beijing, 100000 China

**Keywords:** Computer science, Software

## Abstract

Image stitching is a fundamental pillar of computer vision, and its effectiveness hinges significantly on the quality of the feature descriptors. However, the existing feature descriptors face several challenges, including inadequate robustness to noise or rotational transformations and limited adaptability during hardware deployment. To address these limitations, this paper proposes a set of feature descriptors for image stitching named Lightweight Multi-Feature Descriptors (LMFD). Based on the extensive extraction of gradients, means, and global information surrounding the feature points, feature descriptors are generated through various combinations to enhance the image stitching process. This endows the algorithm with formidable rotational invariance and noise resistance, thereby improving its accuracy and reliability. Furthermore, the feature descriptors take the form of binary matrices consisting of 0s and 1s, not only facilitating more efficient hardware deployment but also enhancing computational efficiency. The utilization of binary matrices significantly reduces the computational complexity of the algorithm while preserving its efficacy. To validate the effectiveness of LMFD, rigorous experimentation was conducted on the Hpatches and 2D-HeLa datasets. The results demonstrate that LMFD outperforms state-of-the-art image matching algorithms in terms of accuracy. This empirical evidence solidifies the superiority of LMFD and substantiates its potential for practical applications in various domains.

## Introduction

In the realm of image stitching, numerous studies have focused on developing feature point detection and description algorithms. Notable algorithms in this domain include feature point detectors such as FAST (Features from Accelerated Segment Test)^1^, SIFT (Scale-Invariant Feature Transform)^2^, SURF (Speeded-Up Robust Features)^3^, and ORB (Oriented FAST and Rotated BRIEF)^4^ as well as feature descriptors such as BRIEF (Binary Robust Independent Elementary Features)^5^ and LBP (Local Binary Patterns)^6^. Among these, FAST-based feature point detection algorithms have exhibited remarkable success in various applications involving visual feature detection, including image stitching, target recognition, and visual mapping. Nevertheless, although attempts have been made to combine FAST with specific feature descriptor algorithms to address its directionality deficiencies, these solutions fail to meet the demanding requirements of real-time performance and convenience in many hardware devices, particularly low-power devices such as cell phones^7^.

This paper introduces a set of descriptors called Lightweight Multi-Feature Descriptors (LMFD), which is designed to enhance the accuracy and efficiency of image matching. LMFD employs a comprehensive approach based on the extraction of gradient information, numerical information, and global information at feature points from which to construct multiple feature descriptors, thereby improving the image stitching process. The feature descriptors constructed in this study are represented as binary matrices consisting exclusively of 0s and 1s. This binary representation facilitates efficient computation through matrix operations, thereby enhancing the algorithm?s suitability for hardware deployment.

**Figure 1:**
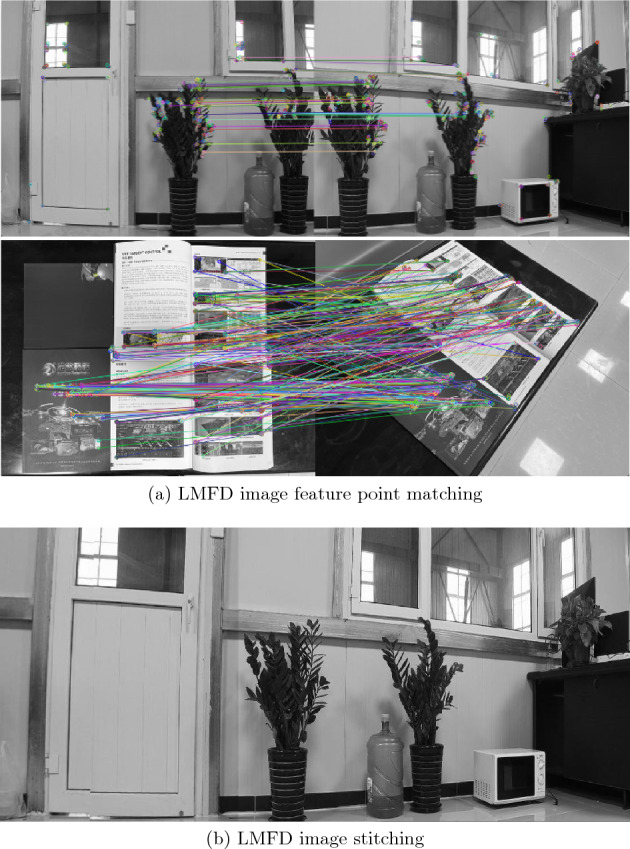
Image feature point matching and image stitching based on LMFD.

Figure 1a depicts the application of the LMFD algorithm to various scenes for feature matching, illustrating its effectiveness. The top panel of Fig. 1a illustrates the feature point matching results in two parallel views featuring numerous similar targets. The results demonstrate that LMFD accurately establishes the matching relationships between feature points, even in the presence of similar targets. This highlights the algorithm?s ability to handle challenging scenarios with a high degree of similarity. In the lower panel of Fig. 1a, the effect of feature point matching in two rotating views containing complex targets is showcased. Despite the complexity and spatial rotation of the targets, LMFD exhibits robust matching capabilities. This demonstrates the algorithm?s resilience and effectiveness in challenging scenarios with intricate and rotated targets. To further validate the efficacy of LMFD, a series of experiments have been conducted to compare its performance with that of other high-quality feature descriptors. These experiments aim to assess the matching capability of the descriptors and their performance in various image matching applications. The results of these comparative experiments provide empirical evidence of the effectiveness and superiority of LMFD.

Figure 1b shows the effect of image stitching after LMFD generation. The image stitching method is similar to that in paper^8^. Specifically, LMFD features are first extracted, and then image registration is performed to find their corresponding relationships in multiple images. Then, the matched feature points are aligned for fusion into the same coordinate system, and after a certain degree of colour correction, the images are finally merged together. The reason why LMFD is highly suitable for image stitching is because its flexible threshold selection and pixel-level high-order vector generation methods can effectively match real image information and provide a reference for feature point alignment. From this figure, it can be seen that seamless connections are achieved for almost all target objects and similar objects, demonstrating the excellent performance of the feature points generated by the proposed algorithm and its corresponding matching method for image stitching.

## Related work

FAST has established itself as a prominent feature point detector renowned for its real-time keypoint detection and visual feature matching capabilities. Its exceptional performance can be attributed to its efficient and convenient pixel-level detection approach. The remarkable speed of detection exhibited by FAST has led to its wide adoption in computer vision and robotics applications. It has proven particularly valuable in scenarios involving high-speed image sequences, such as real-time video streams. Notable advancements include Superpoint^9^, GIC (Geometric Image Correspondence)^10^, and other approaches that leverage neural network methods for keypoint determination. By incorporating neural network techniques, these enhancements aim to refine the keypoint detection process and improve the algorithm?s performance. Superpoint introduces self-supervised learning, which eliminates the need to manually mark points of interest in the training data, thus reducing the cost and complexity of data labelling. However, self-supervised learning methods are highly dependent on large-scale datasets and require sufficient images to learn effective features, and these requirements limit their rapid deployment on terminal devices. Therefore, some researchers have proposed methods of using only a small number of training images to complete registration, such as SIFT Flow^11^, PatchMatch^12^, Patch2Pix^13^ and other methods. The Patch2Pix method uses pixel-level geometric constraints to reduce matching errors and improve matching accuracy. This helps establish accurate correspondence between images from different viewing angles. However, this algorithm may still face some challenges when dealing with low-texture areas or occlusion, leading to inaccuracies in matching.

Although a pixel-level algorithm such as FAST can improve the speed of feature point detection, this type of algorithm is prone to lack directionality in feature detection, making it less effective in scenes where the feature direction is important, and can suffer from false detection issues when faced with poor image quality. To address these limitations and improve the detection performance of FAST and related algorithms, several improved algorithms have been proposed. For example, SIFT and SURF have demonstrated notable outcomes by incorporating direction descriptors to attribute an orientation to each feature point. By introducing directionality, these algorithms have achieved improved results in feature detection. Another notable algorithm is FAST-ER^14^. This algorithm introduces a nonmaximum suppression step into the FAST algorithm, effectively mitigating the problems associated with repeated detection and false positives. By incorporating nonmaximum suppression, FAST-ER eliminates the issue of redundant keypoint detection, improving the efficiency and accuracy of feature detection.

The significance of FAST and its subsequent improvements lies in their ability to enhance the speed and accuracy of feature point detection and matching in computer vision. These advancements enable computers to more efficiently perform tasks such as object recognition, tracking, and 3D reconstruction^15^. Moreover, the practical applications of these algorithms extend beyond computer vision to find relevance in areas such as robotics, where tasks such as autonomous positioning and navigation demand robust feature detection methods.

Regarding feature point descriptors, the SIFT and SURF algorithms employ Gaussian difference operators and a scale-space approach to extract feature points at various scales and rotations. However, these algorithms incur high computational complexity and demand substantial computational resources^16^. In contrast, the BRIEF algorithm adopts binary descriptors to represent feature points, enabling faster feature matching. Additionally, several improvements to the BRIEF descriptor have been proposed, such as rBRIEF^17^, in which randomly selected point pairs are arranged in order of their variance from large to small to select the globally optimal point pair sequence, thus improving the computational efficiency of feature point extraction. However, for each key point, the globally optimal point pair sequence is not necessarily the most suitable. Moreover, various advancements have been made to the BRIEF descriptor^18^, resulting in improved efficiency and computational performance. Examples include the rBRIEF algorithm and other approaches that expedite the feature point extraction process while maintaining high computational efficiency. BOLD (Binary Online Learning Descriptor)^19^ generates multiview images for patches near each key point and then finds the most stable point pair sequence to improve the accuracy of feature point matching. Similarly, FREAK (Fast Retina Keypoint)^20^ and Creak (Color-based Retina Keypoint Descriptor)^21^ are also binary feature descriptors with small storage requirements and high speed, making them suitable for embedded systems and real-time applications.

Deep learning methods have also been employed in the generation of feature point descriptors, as demonstrated by the CNN-FPE (CNN-based Feature Point Extraction)^22^ method. These approaches utilize the features extracted by convolutional neural networks (CNNs) to generate descriptors suitable for computations on field-programmable gate arrays (FPGAs). In DeepDesc^23^, L2-Net^24^ and TFeat^25^, network training is conducted through comparisons of a global loss function, L2 regularization and a triplet loss function, respectively, so that the generated feature descriptors are directionally invariant and robust to large scenes under most conditions. On a similar basis, GeoDesc uses a geometric consistency loss function to train deep learning feature descriptors to improve their robustness to viewpoint changes and affine transformations. These descriptors exhibit high accuracy and robustness, making them effective for tasks such as image stitching. However, it is important to note that this class of descriptors entails complex computations and is less conducive to hardware implementation than the multi-feature descriptor algorithm proposed in this paper.

## Construction of multiple feature descriptors

This paper introduces a novel approach for constructing multi-feature descriptors comprising a symbolic descriptor, a mean descriptor, and a centroid descriptor. By encoding these three descriptors into a compact representation with a specified parameter *bit_width*, the proposed method achieves feature invariance under various transformations, including rotations, scaling, flipping, and affine transformations. The combination of these descriptors contributes to the robustness and adaptability of the multiple extracted features, ensuring their effectiveness in handling diverse visual conditions.

### LMFD feature point detection and matrix decomposition

**Figure 2:**
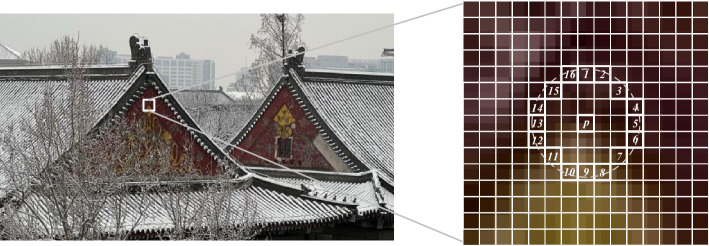
LMFD feature point detection.

As illustrated in Fig. 2, a difference vector $d_{p}$ is calculated within a selected block of the original image, measuring the disparity between the central pixel and its neighbouring pixels. Specifically, for a central pixel $p_{c}$ and its surrounding circular field comprising 16 points, denoted by $p_{n}=1,2,...,15,16$, the difference is computed as $d_{p}=p_{n}-p_{c}$. The resulting difference vector $[d_{0},...,d_{16}]$ captures the local structural characteristics of the image. Notably, this circular field excludes the central pixel, enhancing its robustness against variations in lighting conditions and grey-level changes. Additionally, this difference vector $[d_{0},...,d_{16}]$ offers improved efficiency compared to the original image when applied for feature matching tasks. The difference vector $d_{p}$ can be further decomposed into two distinct components:

1 $$\begin{aligned} {{d}_{p}}={{s}_{p}}*{{m}_{p}}\text { and }\left\{ \begin{matrix} {{s}_{p}}=sign({{d}_{p}}) \\ {{m}_{p}}=\left| {{d}_{p}} \right| \\ \end{matrix} \right. \end{aligned}$$

where $s_{p}$ is the sign component of the difference vector $d_{p}$ expressed as $${{s}_{p}}=\left\{ \begin{matrix} 1\text { }{{d}_{p}}\ge 0 \\ 0\text { }{{d}_{p}}<0 \\ \end{matrix} \right. $$ , and $m_{p}$ is the absolute value component of the difference vector $d_{p}$. The difference vector $[d_{0},...,d_{16}]$ can thus split into a sign vector $[s_{0},...,s_{16}]$ and an absolute value vector $[m_{0},...,m_{16}]$.

**Figure 3:**
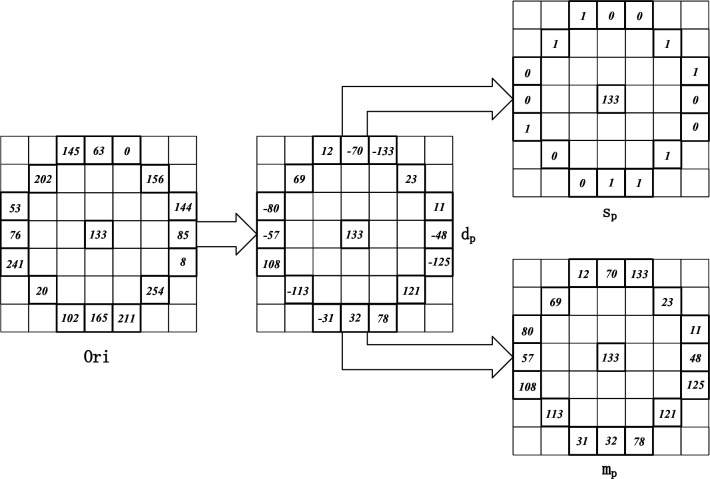
LDSMT transformation of a pixel block.

Reference^26^ introduced the Local Difference Sign-Magnitude Transform (LDSMT), which is based on square field. This transform consists of two components: the sign vector $[s_{0},...,s_{16}]$ and the absolute value vector $[m_{0},...,m_{16}]$. These two vectors are complementary, and the original difference vector can be derived from them. However, in the present paper, a calculation method based on a circular field is adopted instead. As a concrete example, Fig. 3 illustrates the calculation for a pixel block in the original image. The pixel block, denoted by *Ori*, has dimensions of $7\times 7$ pixels. By subtracting the values from the circular field within the pixel block, centred around the central value, from the central pixel value, the difference vector $d_{p}$ is obtained. Subsequently, this vector can be decomposed into the symbol vector [0, 0, 1, 1, 0, 0, 1, 1, 1, 0, 0, 1, 0, 0, 1, 1] and the absolute value vector [70, 133, 23, 11, 48, 125, 121, 78, 32, 31, 113, 108, 57, 80, 69, 12].

The difference vectors calculated in this way partially capture the local image structure. However, using only the difference vectors from a square region as in Local Binary Patterns (LBP) can lead to errors. For instance, consider the two difference vectors $[3,24,-5,66,13,-22,-9,230]$ and $[200,200,-1,200,200,-1,-1,200]$, which share the same sign component $[1, 1, -1, 1, 1, -1, -1, 1]$. Despite the shared sign component, the vector structures are dissimilar. Utilizing these difference vectors directly for image matching would result in significant errors due to the sensitivity of these vectors to variations caused by transformations such as noise, translation, and rotation. The limitations of relying solely on difference vectors for image matching become evident due to this sensitivity. Fluctuations in the feature response stemming from factors such as noise, translation, and rotation will introduce considerable errors when using difference vectors alone.

This paper proposes a decomposition of the difference vector into a sign component and an absolute value component. To determine which component better represents the original characteristic difference, the control variables method is employed to reconstruct the difference signal using only one component. The reconstruction error is then evaluated to determine the component that yields the smaller error. Since the difference signal is the product of the sign and absolute value components, accurate reconstruction cannot be achieved simply through direct removal or setting to zero of a component. Previous research^27^ has established that the difference vector can be modelled using a Laplace distribution. Based on experimental investigations, this paper reveals that the sign component produces smaller errors during the reconstruction process. Accordingly, the obtained sign and absolute value components can be utilized to construct symbolic and mean descriptors, respectively. By decomposing the difference vector and leveraging the optimal component, this approach enhances the accuracy and fidelity of feature representation.

### Symbolic descriptors of LMFD

**Figure 4:**
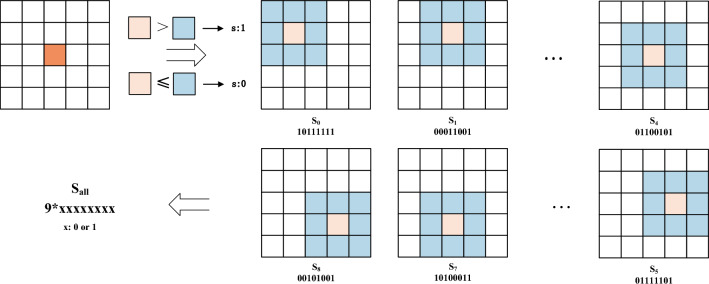
Calculation of symbol descriptor of LMFD.

As an illustrative example, a $5 \times 5$ pixel block is selected around a feature point, and a sliding window of size $3\times 3$ is used to scan the block from the top left to the bottom right. Figure 4 depicts this process. During the calculation of the symbol descriptors, the value of the central pixel within each sliding window is compared to the value of each surrounding pixel within the window. If the central pixel value is greater than the surrounding pixel value, the corresponding surrounding position in the descriptor is set to 1; otherwise, it is set to 0. The formula representing this process is as follows:

2 $$\begin{aligned} LMFD{{S}_{n,t}}=\sum \limits _{n=0}^{N-1}{{{s}_{p}}\cdot {{2}^{p}}},{{s}_{p}}=\left\{ \begin{matrix} 1\text { }{{d}_{p}}>0 \\ 0\text { }{{d}_{p}}\le 0 \\ \end{matrix} \right. \end{aligned}$$

Where *N* is the number of scans of the sliding window, and *p* is the number of sliding window domains around the feature point, and $s_{0},s_{1},...,s_{7},s_{8}$ are calculated in turn, and $s_{all}$ is obtained by arranging and combining $s_{0}$ to $s_{8}$ in order to generate the symbolic descriptor.

### Mean descriptor of LMFD

**Figure 5:**
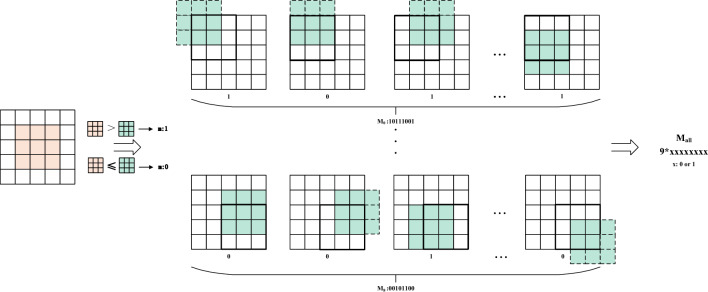
Calculation of mean descriptor of LMFD.

The mean descriptor is subsequently computed within the same selected pixel block, utilizing a sliding window of the same size and movement pattern as that employed in the calculation of the symbolic descriptor. Figure 5 illustrates this process. The mean value of the pixels within the sliding window in which the feature point is located is compared to the mean value of all pixels within each sliding window. If the mean value of the feature point window is greater than the mean value of the current sliding window, the value at the corresponding position in the descriptor is set to 1. Otherwise, if it is less, then the descriptor value at this position is set to 0. The formula representing this calculation is as follows:

3 $$\begin{aligned} LMFD{{M}_{n,t}}=\sum \limits _{n=0}^{N-1}{f({{m}_{p}},{{m}_{c}})\cdot {{2}^{p}}},f(x,{{m}_{c}})=\left\{ \begin{matrix} 1\text { }x>{{m}_{c}} \\ 0\text { }x\le {{m}_{c}} \\ \end{matrix} \right. \end{aligned}$$

### Centroid descriptor of LMFD

**Figure 6:**
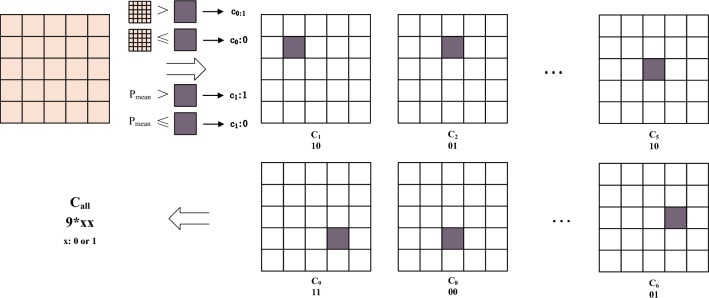
Calculation of centroid descriptor of LMFD.

The calculation of the centroid descriptor follows a similar approach. As depicted in Fig. 6, a sliding window consisting of nine pixels is employed, scanning from the top left to the bottom right. The magnitude of the centroid value within each sliding window is compared to the average value of the pixel block surrounding the selected feature point as well as the average value of the entire image. If the magnitude of the centroid value is greater than the average value, the value at the corresponding position in the descriptor is set to 1; otherwise, it is set to 0. The formula representing this calculation is as follows:

4 $$\begin{aligned} LMFD{{C}_{n,t}}=\sum \limits _{n=0}^{N-1}{f({{m}_{p}},c)\cdot {{2}^{p}}} \end{aligned}$$

### Combination of multiple feature descriptors

**Figure 7:**
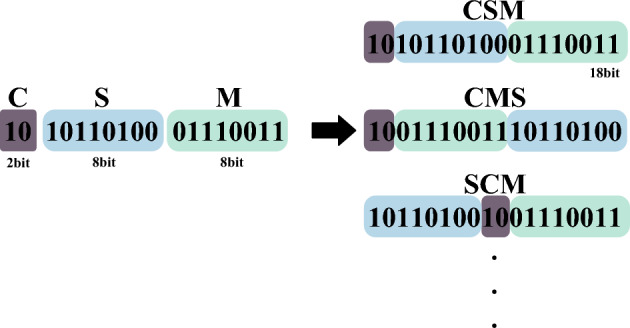
Different combinations of the three descriptors.

Once the above three descriptors have been generated, this paper explores their effects in different combinations to find the optimal combination that provides the best image matching results. This paper first considers directly splicing all three binary descriptors together with different splicing methods depending on the importance of each of the three descriptors. As shown in Fig. 7, the 18-bit multiple descriptors consisting of the symbolic, mean and centroid descriptors are grouped from high to low to form a total of six combinations, denoted by CSM, CMS, SCM, SMC, MSC and MCS, where C denotes the centroid descriptor, S denotes the symbolic descriptor, and M denotes the mean descriptor. In this paper, the multiple feature descriptors generated via the above six combinations are each tested for image matching, and the test results are reported in Section 4.5. It is found that feature point matching tends to yield better results when the mean descriptor M is placed at a high level, consistent with the findings presented in paper^28^.

The pseudocode of the multiple feature descriptor-based image matching algorithm is shown in Algorithm 1. The input consists of two images to be stitched together, I1 and I2; the parameter r, which is used to control the radius of the detection region around each detected feature point; the convolution kernel size, kz; and the parameter ps, which controls the image patch size around each detected feature point. The output is the stitched image. The algorithm proposed in this paper first processes the image with a greyscale gradient to facilitate subsequent feature extraction. The greyscale image is then subjected to feature point extraction. For this purpose, the block of image pixels around each feature point is extracted with a radius of ps around the location of the feature point. The symbolic descriptor, the mean descriptor and the centroid descriptor are calculated for all points in this pixel block at a radius r from the feature point, and the three descriptors are then combined into a multi-feature descriptor in accordance with a selected combination method. Based on these multi-feature descriptors, the feature points are matched, and the images are stitched together.

**Algorithm 1:**
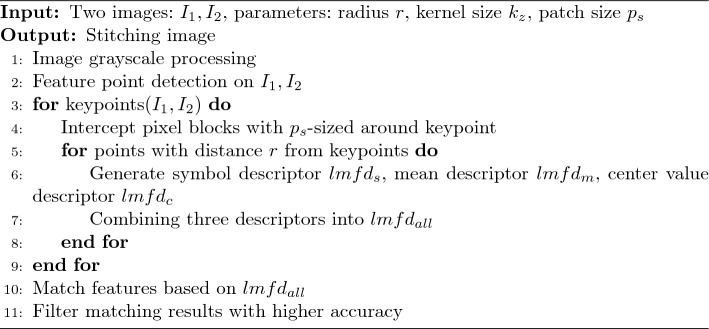
Image stitching algorithm based on multiple feature descriptors.

## Experiments

The algorithms proposed in this study were evaluated on a workstation with the following specifications: an Intel Core i7-8700K processor (CPU) with a clock speed of 3.7 GHz, 6 cores, and 12 threads, accompanied by 8 GB of DDR4 2400 MHz RAM. The graphics processing unit (GPU) used was an NVIDIA GeForce GTX 1080 Ti with 11 GB of GDDR5X memory. The algorithm implementation was conducted using Python 3.6 and OpenCV 3.4.2.16.To assess the performance of the proposed algorithms, two publicly available datasets were employed for validation. The first dataset is Hpatches (Homography Patches)^29^, which serves as a benchmark for evaluating the robustness and accuracy of image matching algorithms. The second dataset is 2D-HeLa^30^, which provides a valuable evaluation platform for image matching techniques.

### Experiments on image feature point matching

**Figure 8:**
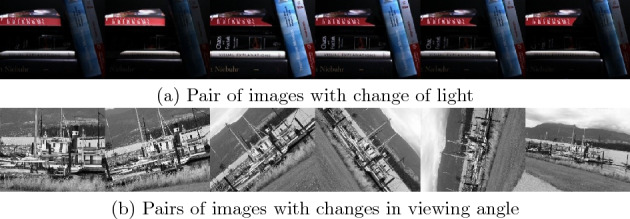
Image pairs in the HPatches dataset.

HPatches is an extensive dataset specifically designed for assessing the performance of local descriptors. It comprises images of 116 types, with 57 corresponding to detection pairs featuring variations in lighting conditions, and 59 corresponding to detection pairs with viewpoint changes, as depicted in Fig. 8. The dataset’s key attributes, including its reproducibility, diversity, origin in real data, large scale, and multitask nature, establish it as an objective benchmark for evaluating the effectiveness of local descriptors. Researchers widely employ HPatches to objectively gauge the performance of their local descriptor algorithms.

This paper focuses on evaluating the performance on HPatches specifically in the context of image stitching. The experimental setup follows the guidelines proposed by the authors of D2Net^31^. The evaluation metrics employed include the mean matching accuracy (MMA) at thresholds ranging from 1 to 10 pixels as well as the numbers of matches and features extracted from the images.

**Figure 9:**
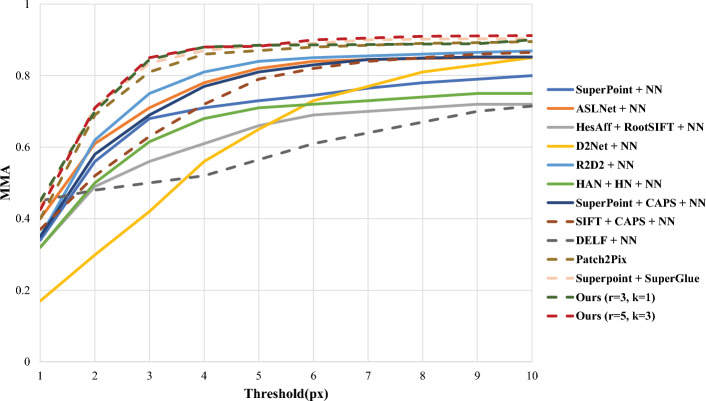
Comparison of average matching accuracy under thresholds ranging from 1 to 10 pixels on HPatches.

Regarding the experimental setup, confidence scores from the fine-level regressor are employed in this study to filter out outliers. The aim is to strike a balance between match quantity and quality by dynamically adjusting parameters such as the convolution kernel size $k_{z}$ and the search radius around feature points. In addition to comparing the proposed method with various local feature methods that employ nearest neighbour (NN) searches for matching, this paper also evaluates SuperPoint features matched with SuperGlue^32^. The experimental results, including comparisons and performance metrics, are presented in Fig. 9 and Table 1.

Figure 9 shows the MMA values at thresholds ranging from 1 to 10 pixels. Weakly supervised method are represented with dashed lines, and fully supervised methods are represented with solid lines. From this figure, it can be seen that most algorithms exhibit slow and stable MMA growth after the pixel threshold reaches 4. LMFD refines the patch-level matching commonly used in other algorithms to pixel-level correspondence, greatly improving the matching accuracy under viewpoint changes and further improving the matching accuracy under lighting changes. When LMFD is compared with all weakly supervised methods, our model performs best with parameters of $r=3,k_{z}=1$ . When the pixel threshold is less than or equal to 5, the performance of SuperPoint+SuperGlue^32^ is similar to that of our model. However, our algorithm outperforms all other fully supervised methods.

**Table 1:** Analysis of the number of feature point detections versus the number of matches on the HPatches dataset.

Methods	Features/Matches
Superpoint^15^+NN	2.0K/1.1K
ASLNet^33^+NN	4.0K/2.0K
HesAff^34^+RootSIFT+NN	6.7K/2.8K
D2Net^31^+NN	6.0K/2.5K
R2D2^35^+NN	5.0K/1.6K
HAN^36^+HN+NN	3.9K/2.0K
Superpoint+CAPS^37^+NN	2.0K/1.1K
SIFT+CAP^38^+NN	4.4K/1.5K
DELF^39^+NN	4.6K/1.9K
Patch2Pix^13^	2.4K/1.1K
Superpoint+SuperGlue^32^	1.1K/0.5K
Ours($r=3,k_{z}=1$)	3.6K/2.1K
Ours($r=5,k_{z}=3$)	2.5K/1.6K

Based on Fig. 9, Table 1 further illustrates the performance of various algorithms, including SIFT, D2Net, and HesAff, in terms of feature point detection and matching under changes in illumination and viewpoint. While these algorithms demonstrate a greater ability to detect feature points, their relative numbers of successful matches are comparatively low. This discrepancy arises from the limitations inherent in SIFT-based image matching, which struggles to accurately extract features and descriptors for targets with smooth edges and exhibits poor rotational invariance^40^. Section 4.2 specifically evaluates this aspect, revealing suboptimal pixel-level matching performance when handling images with varying illumination conditions or viewing angles.

The LMFD feature descriptors proposed in this paper are combined with FAST feature detection. LMFD is used to establish a direct correspondence between the 0/1 matrices of the feature descriptors and the image pixels, greatly improving the matching accuracy under viewpoint changes and further improving the matching accuracy under illumination changes. When the parameters are set to$r=3,k_{z}=1$, the number of matching points can reach 2.1K based on the 3.6K detected feature points. When the parameters are set to $r=5,k_{z}=3$, the detection area around each feature point is larger, making the nonextreme value phenomenon around the feature points more obvious; thus, the number of detected points drops to 2.5K, but the number of matching feature points is still 1.6K. Accordingly, the degree of matching on the HPatches dataset is better with our algorithm than with the other algorithms mentioned above.

### Rotation invariance of feature descriptors during matching

**Figure 10:**
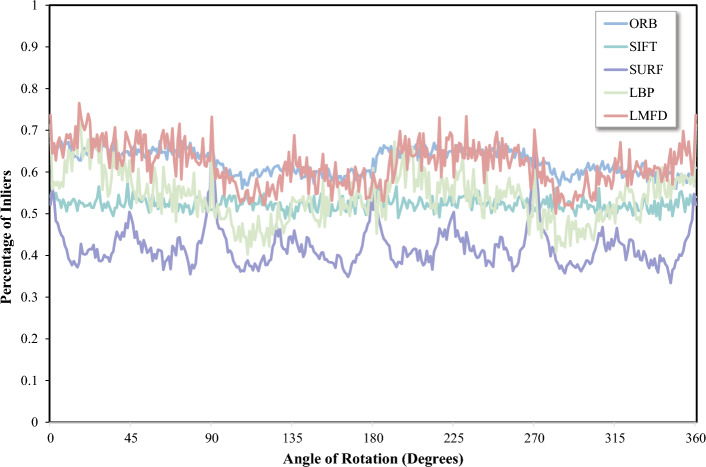
Comparison of image matching of various algorithms under image rotation.

To assess rotation invariance, tests are conducted on the HPatches dataset. The average number of matches is compared across rotations ranging from $0^{\circ }$ to $360^{\circ }$ for the 59 image pairs with perspective changes in this dataset . The results, depicted in Fig. 10, demonstrate that the algorithm proposed in this paper outperforms other algorithms such as SIFT, SURF, and ORB in terms of matching performance. Notably, SURF exhibits lower overall matching performance in this test for matching with image rotation. Furthermore, a quantized reflection phenomenon occurs approximately every $45^{\circ }$ starting at $0^{\circ }$ for SURF, leading to a less stable feature point matching process^41^. The superior matching degree of the algorithm proposed in this paper demonstrates its robustness and effectiveness in handling image rotations.

### Noise invariance of feature descriptors during matching

**Figure 11:**
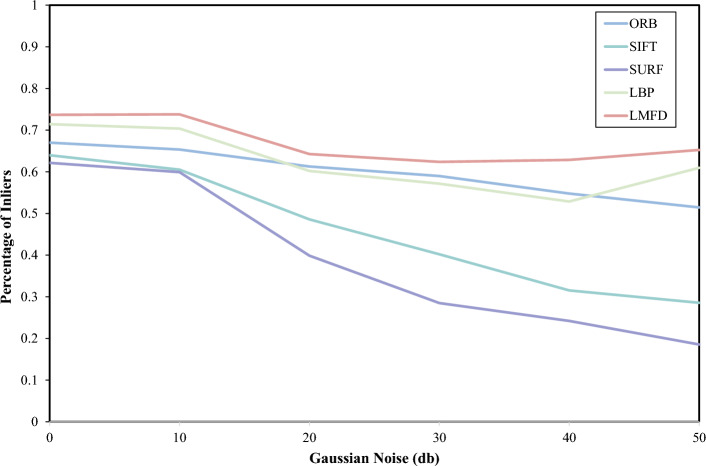
Comparison of image matching degree of various algorithms under Gaussian noise.

To evaluate the noise immunity of the proposed algorithm, this study compares the matching ratios before and after the introduction of various levels of Gaussian noise into the 57 pairs of images with lighting changes in HPatches. The results are presented in Fig. 11. The algorithm proposed in this paper outperforms algorithms such as SIFT and SURF across all levels of Gaussian noise, exhibiting superior matching results. Compared to ORB, the proposed algorithm demonstrates better performance in image matching at higher noise levels, highlighting its enhanced robustness to noise in image matching scenarios. Although ORB performs well at lower noise levels, the proposed LMFD algorithm exhibits a stronger noise robustness effect as the noise level increases.

**Figure 12:**
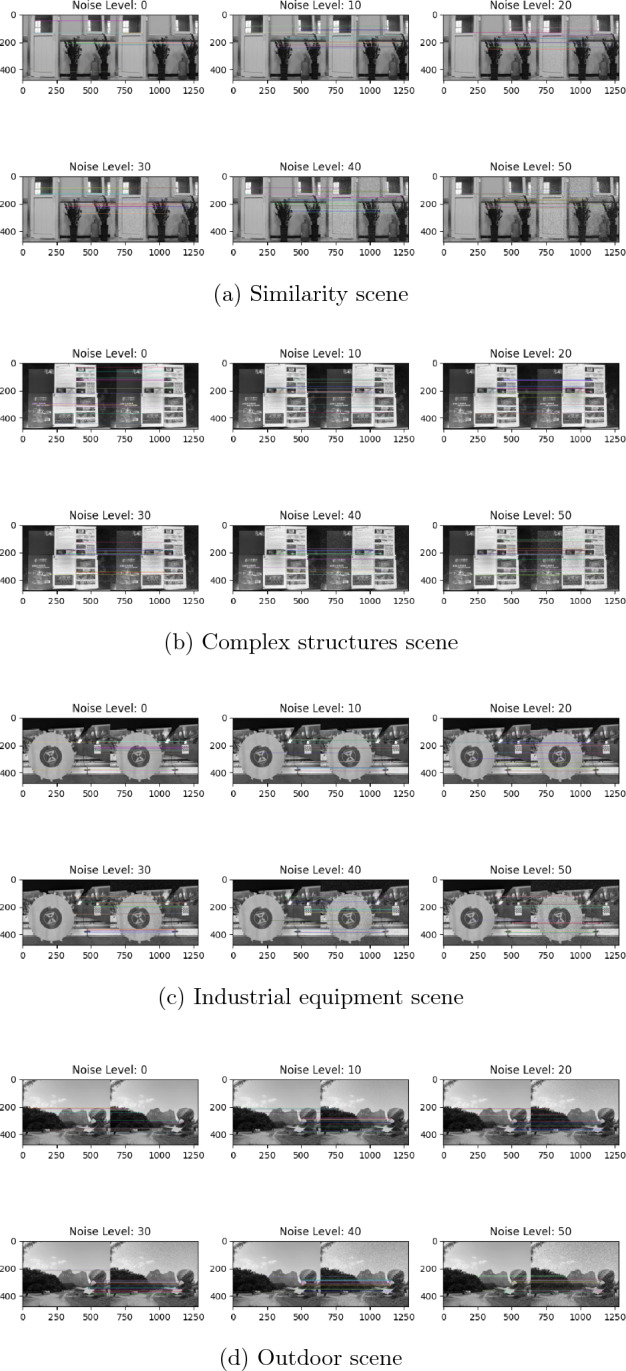
The effect of LMFD in matching feature points under various Gaussian noise levels.

To further demonstrate the robustness of LMFD in feature point matching, examples of the results of the feature matching experiments across a range of Gaussian noise levels from 10 to 50 are visualized in Fig. 12. The selected examples represent four common scenarios in daily life, including a scene with many similar objects, a scene with complex structural components, a scene with industrial equipment, and a scene representing an outdoor space. From this figure, it can be seen that LMFD exhibits good feature point matching performance under increasing Gaussian noise in all four scenarios. Especially in scenes with many similar objects and complex structures, the matching quantity and accuracy of the feature points are high thanks to the pixel-level window scanning process in LMFD, which allows it to more accurately extract high-order features from images. These results also demonstrate the accuracy and reliability of LMFD under various levels of Gaussian noise.

### Effects of different combinations of multiple feature descriptors

**Figure 13:**
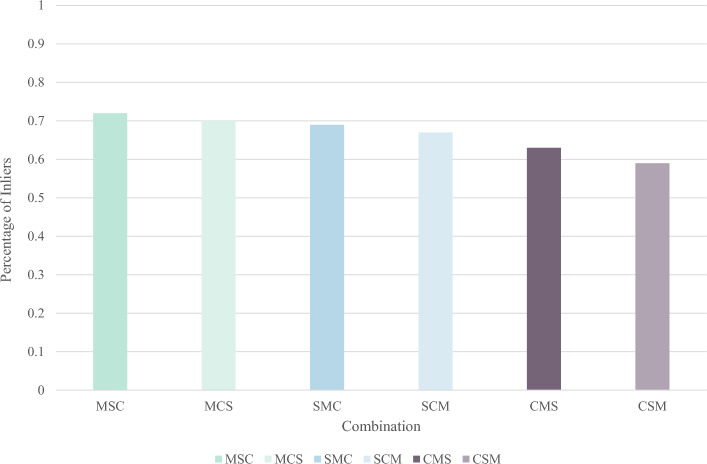
Matching degree comparison for different combinations of descriptor permutations in LMFD.

This section examines the matching performance of the mean, symbolic, and centroid feature descriptors in differently permuted combinations. Six combinations, namely, MSC, MCS, SMC, SCM, CMS, and CSM, are tested based on the arrangement of high and low bits within the feature descriptors. As seen in Fig. 13, the results indicate that placing the mean descriptor at a higher bit count leads to improved feature descriptor matching. This can be attributed to the fact that the mean descriptor captures more comprehensive feature information during the convolution process than the symbolic and centroid descriptors do. It is crucial to carefully control the size of the convolution kernel to ensure maximum coverage of the information around feature points while maintaining computational efficiency.

### Computation times of LMFD and other feature descriptors

This section compares the extraction time and the quantity of extracted feature points between LMFD and several other algorithms. The data are selected from a random set of 100 images in the Hpatches dataset, and the various algorithms are applied to this set of images to compare the number of feature points extracted and the time needed for extraction. Table 2 shows the number of feature points and the time of feature point detection for LMFD and the other algorithms. From the table, it can be seen that for the algorithm proposed in this paper, when the parameters are set to $r=3,k_{z}=1$, although the number of detection points is not as high as with FAST, the detection time is slightly shorter, with an average time per image of 2 ms. When the parameters are set to $r=5,k_{z}=3$, although the detection time increases, the quality of the feature points is significantly improved.

**Table 2:** Comparison of the number and time of feature point extraction.

Methods	Time (ms)	Features
SURF^3^	176	2911
FAST^1^	2	5158
BRISK^42^	10	1874
DoG^43^	338	1552
ORB^4^	7	594
Ours($r=3,k_{z}=1$)	1.87	2513
Ours($r=5,k_{z}=3$)	10.35	648

This article also reports a similar experiment on the computation times of various descriptors. For 1000 feature points extracted by SURF, the time consumed by LMFD and several other algorithms for point matching is compared, and the results are summarized in Table 3. Generally, as the number of features increases, the matching time increases approximately linearly. From this table, it can be seen that although LMFD is slower than BRIEF, it is still faster than most other algorithms. Moreover, for high-quality feature points that can yield better results in subsequent practical applications such as image stitching, the matching speed of the algorithm proposed in this article still approaches the leading level.

**Table 3:** Comparison of computation times of the different descriptors.

Methods	Time (ms)
SURF^3^	117.1
BRISK^42^	10.6
BRIEF^5^	3.8
ORB^4^	4.2
SIFT^2^	448.6
LIOP^44^	1801.1
MROGH^45^	2976.8
Ours($r=3,k_{z}=1$)	4.1
Ours($r=5,k_{z}=3$)	12.3

### Applicability for texture feature classification

To showcase the versatility of LMFD in image representation, this paper reports the application of the proposed algorithm to a medical image analysis task, specifically texture feature classification. This task is highly relevant because texture classification plays a crucial role in biomedical diagnostics. The 2D-HeLa dataset, widely used as a benchmark in the field, is employed to evaluate the ability of feature descriptors to classify texture features^46^. To ensure fair comparisons, adaptive histogram equalization with limited contrast is utilized, and a radial basis function kernel SVM is employed, similar to the approach used in LBP. This experimental setup allows for a comprehensive assessment of LMFD?s performance in texture feature classification, highlighting its effectiveness and applicability in diverse image analysis tasks.

The 2D-HeLa dataset serves as a valuable resource for protein cell classification, specifically the automated identification of subcellular organelles in fluorescence microscopy images. This dataset comprises a total of 862 images, with each category containing 70–98 images^47^. The resolution of all images in the 2D-HeLa dataset is $382 \times 382$. Due to the nonrigid motion of HeLa cells, the images exhibit significant variations in appearance.

In this study, a comparative analysis is conducted between the proposed LMFD method and other state-of-the-art techniques on the 2D-HeLa dataset. The techniques considered for comparison include popular methods such as LBP, LPQ (Local Phase Quantization)^48^, LTP (Local Ternary Patterns)^49^, MRD (Multiscale feature fusion and Reverse attention network for Detection)^50^, and disCLBP (Discriminative Completed Local Binary Patterns)^51^. The average accuracy in more than 5-fold cross-validation is also evaluated using a suitable metric^52^. The results, presented in Table 4, demonstrate that the method proposed in this paper achieves an impressive accuracy of 95.9%, surpassing the performance of all other compared methods. Table 4 also compares the average classification accuracy and standard deviation results of LMFD and other algorithms. It can be seen that LBP and SAHLBP^53^, relative to LMFD, exhibit significantly different performance on the 2D-HeLa dataset despite their pixel-level feature descriptor construction. The reason is that LMFD uses sliding windows to construct omnidirectional triple descriptors for local pixels, while the other two algorithms only analyse the arrangement and combination of single-pixel blocks. This leads to differences in their ability to capture subtle differences between categories, thereby widening the gap in average accuracy. This finding further underscores the effectiveness and superior performance of the proposed LMFD algorithm in protein cell classification tasks.

### Ethical and informed consent

The dataset used in this article has obtained permission from relevant institutions and patients, and has been publicly published. There are no issues with data ethics.

**Table 4:** Comparison of feature classification accuracy of feature descriptors on 2D-HeLa dataset.

Descriptors	Accuracy (%)
LBP^10^	$82.3 \pm 2.13$
LPQ^48^	79.70 ± 2.55
SAHLBP^53^	84.49 ± 2.21
CNN-LCC^54^	90.72 ± 2.17
DLTP^49^	92.21 ± 1.53
MRD^50^	95.34 ± 1.99
disCLBP^51^	95.45 ± 2.20
Ours($r=5,k_{z}=3$)	95.96± 2.16

## Conclusions and future work

This paper presents LMFD, an algorithm based on lightweight multiple feature descriptors, for image stitching. Unlike traditional methods that rely on a single feature descriptor, LMFD incorporates symbolic, mean value, and centroid information around feature points and organizes this information into a binary matrix format. By combining these descriptors, the algorithm simplifies computations while maintaining robust matching performance in the presence of illumination, rotation, and noise variations. Experimental results also demonstrate that LMFD achieves superior texture classification accuracy compared to existing algorithms. The proposed LMFD algorithm thus offers a promising approach for enhancing performance in image stitching and texture classification tasks.

Despite the advancements enabled by the algorithm proposed in this paper, there are still opportunities for further improvement. One avenue that could be pursued is the optimization of the computation time. Currently, redundant calculations are performed when constructing the multiple feature descriptors, leading to repetitive computations for points surrounding the same feature point. Streamlining these calculations would improve the algorithm?s computational efficiency. Furthermore, the parameter adjustment in this paper could be further optimized. For instance, the selection of parameters such as the detection radius around feature points and the size of the convolution kernel for descriptor calculation could benefit from neural network training to achieve better performance. Optimizing these parameters represents a promising direction for future research and development. By addressing these areas for improvement, the algorithm’s overall effectiveness and efficiency could be enhanced.

### Supplementary Information

Supplementary Information.

## Data Availability

The datasets and code used during the current study are available from the “LMFD.rar” compressed file submitted in this manuscript, or it can be obtained from the corresponding author on reasonable request (Supplementary information).
